# The Outcome of Immediate Administration of Dexamethasone in Children With Croup (Laryngotracheobronchitis) in King Abdullah Specialized Children’s Hospital

**DOI:** 10.7759/cureus.25726

**Published:** 2022-06-07

**Authors:** Abdulaziz A Alqahtani, Nazish Masud, Mohammad S Algazlan, Saleh S Alqarni, Khalifah N Almutairi, Abdullah A Bahumiad, Sulaiman A AlQueflie

**Affiliations:** 1 College of Medicine, King Saud Bin Abdulaziz University for Health Sciences, Riyadh, SAU; 2 Research Unit, College of Medicine, King Saud Bin Abdulaziz University for Health Sciences, Riyadh, SAU; 3 Research, King Abdullah International Medical Research Center, Riyadh, SAU; 4 Pediatrics, King Abdulaziz Medical City Riyadh, Riyadh, SAU

**Keywords:** croup, laryngo-tracheobronchitis, dexamethasone, pediatric, saudi arabia

## Abstract

Background

Croup is an inflammatory disease that affects the upper respiratory tract involving the upper airways of the lungs (bronchial tubes), vocal cords (larynx), and windpipe (trachea). In Canada, it is considered one of the major causes of respiratory diseases in the first 10 years of life. A wide range of viruses like common cold and flu (influenza) infections can cause croup (laryngotracheobronchitis). Dexamethasone has been commonly used to treat croup even though it lacks evidence on patients' recovery. The study aimed to compare the effect of the immediate or late dexamethasone administration on patient recovery and identify predictors for relapse among children with croup.

Methods

A retrospective cross-sectional study was conducted using the electronic medical record (Best Care) of all croup patients between 2014 and 2018 in King Abdullah Specialized Children's Hospital (KASCH), in Riyadh, Saudi Arabia. Out of the 329, only 186 patients matched our inclusion criteria. Statistical analysis was conducted with the SPSS V.22 software package (IBM Corp., Armonk, NY). The main outcome variable was early recovery or relapse. The chi-square test and logistic regression were used to assess the relationship between the independent variables with recovery or relapse among croup patients. A p-value of <0.05 was used to determine the significance of the test.

Results

Fifty-three recovered out of the 186 patients. Moreover, 50 of the recovered patients were treated in the ER. In addition, out of the 53 patients who recovered, 40 patients were treated as inpatients (IPs). Those who were given dexamethasone immediately for both recovery and relapsed groups were 29% and 71%, respectively while those who were given dexamethasone late were 34% for the recovery group. On the other hand, 119 patients relapsed. Out of those patients who relapsed, 111 were treated in the ER. Moreover, out of relapsed patients, 79 patients were treated as IPs. Furthermore, out of 186 patients, 86 had chronic illnesses. Twenty-four percent (24%) of those with chronic illnesses recovered, and 76% relapsed (P-value=0.04). Also, there was a significantly higher trend of administration of dexamethasone immediately in the ER in 69% of children with a p-value <0.001.

Conclusion

In conclusion, the difference between the early and late administration of dexamethasone in both recovery and relapse is not significant. Also, the presence of chronic illnesses affects relapses more significantly.

## Introduction

Croup is an inflammatory disease that affects the upper respiratory tract involving the upper airways of the lungs (bronchial tubes), vocal cords (larynx), and windpipe (trachea) [1]. Croup, almost always, occurs in late autumn and early winter [1]. In Canada, it is considered one of the major causes of respiratory diseases in the first 10 years of life [2]. A total of 80,000 Canadian children get affected by croup annually [2]. It is known globally that boys are at higher risk than girls (1.4:1) [2]. A population study conducted from 1999 to 2005 found that 94.4% of children with croup did not require admission [3]. Children aged three years old or younger are more susceptible to developing croup since they have small airways, but it can also occur in children who are older [4]. A wide range of viruses like common cold and flu (influenza) infections can cause croup (laryngotracheobronchitis), and it tends to be, irregularly, caused by Mycoplasma pneumonia [1,4]. Almost 75% of the total cases are caused by parainfluenza, with parainfluenza type 1 being the most common subtype [4]. The rest of the cases, according to prospective cohort studies, are typically caused by respiratory syncytial infection, influenza A and B, adenovirus, rhinovirus, and mycoplasma [4]. Occasionally, a bacterial infection might cause croup [1]. A large number of children who are infected with viruses that can cause croup will, generally, develop cold symptoms and not croup [1]. Other causes include allergies and reflux [4].

There is no difference in the pathogenesis of croup regardless of which virus causes it. Initially, the pathogen infects the nasal and pharyngeal mucosa and then spreads down toward the lower respiratory tract. when the pathogen reaches the subglottic area, The classic croup symptoms are produced by the inflammation and edema that occur in the area, which eventually leads to the narrowing of the respiratory tract. This narrowing on imaging is characteristic of croup. This happens because of the complete ring shape of the cricoid cartilage, which is unlike the tracheal cartilage that is C-shapes. Additionally, a child's mucosa is floppy, so whenever it cries or becomes agitated, it causes a dynamic obstruction [5-6].

Clinically, the three typical symptoms of croup are inspiratory stridor, hoarseness, and cough. Barking cough is the most common symptom for children while hoarseness is the most common symptom for adults. Symptoms of croup, caused by the parainfluenza virus, are initially nonspecific and difficult to diagnose but mild and thus do not require urgent medical treatment. In the beginning, it is characterized by nasal congestion and discharge, which last usually around two days, and then it develops into fever and the classic symptoms of croup. Typically, the cough resolves faster within days, unlike the other symptoms that could remain for a longer duration [7]. The physician should think about other possible diagnoses if the clinical presentation of the patient is different from the classical symptoms. Usually, a more severe disease is expected in cases of patients presenting with atypical symptoms. In addition, there are other factors that may contribute to the severity of the disease, for example, if the disease develops fast and suddenly, especially under 12 hours, if the patient has had the same infection before, and when there is an abnormality in the respiratory tract or any diseases that can aggravate the disease into severe forms like asthma [5].

When reaching the diagnosis clinically, patients need to be assessed properly to approach management in the best way. There are known various scoring systems for this purpose. The most famous one is the Westley Croup score [5], in which the severity of croup is established by the presence or absence of stridor at rest, the mental status, the presence or absence of pallor or cyanosis, air entry, and the degree of chest wall retractions. Every one of these five criteria has a numerical value, and the disease will be placed into one of four severity groups based on the sum of these values: mild, moderate, severe, or impending respiratory failure.

Finally, patients will be managed depending on the category of severity that they fall under. In general, patients in the mild or moderate classification are treated at home while for those with worse conditions, admission may be needed.

Patients should be evaluated by their physicians at the time of presentation. When a patient has hypoxemia or respiratory distress, oxygen must be provided immediately. In severe disease, corticosteroids are helpful in relieving symptoms and reducing edema in the laryngeal mucosa due to their anti-inflammatory effect [6]. Russell et al. mentioned that the severity score reduced at six and 12 hours after the use of steroids with the reduction of staying time in the ER and return visits [8]. Furthermore, lower doses of epinephrine are required for those who are treated with steroids.

Fernandes et al. found out that using steroids in children who have acute respiratory distress is safe [9]. Dexamethasone is superior to budesonide and prednisolone in the management of croup. Dexamethasone has a diverse route of administration; it can be given as a single dose orally, intramuscularly, or intravenously, with the most commonly used dose being 0.6 mg/kg [10]. Bjornson et al. discussed that the use of epinephrine helps in reducing and controlling the symptoms. It decreases edema by causing arteriole vasoconstriction in the upper airway mucosa. It is advised to use epinephrine along with dexamethasone due to its fast action, but it has a short half-life compared to dexamethasone, which has a late onset of action but a longer half-life. Epinephrine should be administered via nebulizer at a dose of 0.05 milliliters per kilogram of racemic epinephrine 2.25% (maximum dose = 0.5 milliliters) or 0.5 milliliters per kilogram of L-epinephrine 1:1,000 (maximum dose = 5 milliliters) [11-12].

To our knowledge, there are no local studies in Saudi Arabia about this objective, however, globally, there is a similar study in Canada. They compared the patients who took prehospital dexamethasone and those who did not take prehospital dexamethasone. Those who took prehospital dexamethasone have less requirement for another drug with 5% against 32.7% for the other group. And in the case of length of stay at the hospital, they were also less, with an average of 2.6 hours. But in the case of both admission and relapse, those who did not take prehospital dexamethasone had better outcomes, with 3.5% and 6.5%, respectively, against 10% for both variables in the other group.

There is a need to assess the clinical impact of early dexamethasone administration on recovery in order to determine if it has a beneficial effect on clinically relevant patient outcomes for children with croup, as well as to reduce both the clinical and financial burden and improve health care services. This research aimed to compare the outcomes between giving the drug (dexamethasone) immediately to children with croup or waiting until further investigation, which is not known.

## Materials and methods

Study settings and participants

A retrospective cross-sectional study was conducted using the electronic medical records (Best Care) of all croup patients between 2014 and 2018. Of the 329, only 186 patients matched our inclusion criteria. The electronic medical records were reviewed to ensure that they were eligible for inclusion. King Abdullah Specialized Children's Hospital (KASCH), Riyadh, is the Kingdom of Saudi Arabia's first specialized children's hospital, with a total area of 192,000 m^2^ divided over 10 floors. All patient files are kept electronically at the hospital, which is a paperless digital hospital. The emergency department, as well as the state-of-the-art daycare, diagnostic, and treatment departments, are located on the hospital's fourth podium. The hospital has 60 beds in the emergency department for pediatric emergencies and trauma.

Our research included all Saudi children (3 months to 3 years) with croup in the hospital and both genders.

We excluded croup patients with a bacterial infection, such as bacterial tracheitis and epiglottitis; croup patients with congenital anomalies, such as great vessel anomalies and laryngeal cleft; non-Saudi patients, and patients younger than three months or older than three years.

All the identified croup patients (329) were reviewed between 2014 to 2018. Of these, 143 medical records were excluded based on our exclusion criteria.

Data collection

The data was retrieved from the Best Care system, an electronic medical records system used by the Ministry of National Guard Health Affairs (MNGHA), under the supervision of a pediatric consultant and a statistician. Medical records information was collected using a prepared data collection form. The students used the prepared data collection form to ensure the consistency of coding. The supervisors reviewed the data collected by the students to ensure the accuracy of the data. All the data were regularly checked for accuracy and the missing information was collected by revisiting the patient records. The medical record numbers were replaced with unique identifiers, maintaining the confidentiality of the participants. The data were kept in a password-protected file, and only the research team had access to it. King Abdullah International Medical Center’s institutional review board approved this study with reference number IRBC/1610/19.

Data analysis

The final sample of 186 patients was included in the data analysis. The data were first extracted in Microsoft Excel (Microsoft Corporation, Redmond, WA) and checked for correctness. Statistical analysis was conducted with the SPSS V.22 software package (IBM Corp., Armonk, NY). The main outcome variable was early recovery or relapse. Results were presented based on the distribution of recovery or relapse among the patient sample. The descriptive statistics were presented as percentages out of the total sample for the categorical variables like gender, mode of admission, presence of chronic illnesses, etc. For numerical variables like age, days of illness, height, and weight, mean and standard deviations were reported in the summary tables. The chi-square test was used to assess the relationship between the independent variables with the recovery or relapse among croup patients. An independent sample t-test was used to assess the difference in the first and second doses of dexamethasone given to children. For predictors of early relapse, the binary logistic regression was applied and an odds ratio along with a 95% confidence interval was reported. As a measure of goodness of fit for logistic regression, the Hosmer-Lemeshow test was used. Recovery was the dependent variable and relapse was considered as ‘1’. For all the categorical variables ‘last,’ was considered as the reference category. A p-value of <0.05 was used to determine the significance of the test.

## Results

Descriptive profile of study participants

A total of 186 patients, ranging from three months to three years, were included in this study. More than two-thirds of the sample (130; 70%) were males and less than one-third were females (56; 30%,). The majority of the patients 167 (94%) were treated in the emergency department. The mean age of the participants was 25±22 months. Overall, 118 (67%) patients (n=118) were given dexamethasone immediately. The most commonly administered route was oral (166; 95%) and then intravenous (6; 3%) and intramuscular 2% (n=3). The admission rate was prominent with a percentage of 125 (71%) (Table 1).

**Table 1:** Profile of the children with croup given immediate or late treatment of dexamethasone (N=186) * Chronic illnesses: diabetes mellitus, bronchial asthma, glycogen storage disease type 1, congenital heart disease, autism, Down syndrome, G6PD, and chronic disease of tonsil and adenoid The number of N/A data for: Mode of birth: 14, Vaccination status: 8, Chronic illnesses: 7, Treated in ER: 9, Admitted: 9, Route of administration: 11, Administration of dexamethasone: 9, Early recovery: 14

Variables	Categories	Mean,	SD
Age in months	Mean, SD	25	22
Weight ( kg)	Mean, SD	10	7
Height (cm)	Mean, SD	77	21
Duration of the illness(days)	Mean, SD	3	2
		Frequency	Percentage
Gender	Male	130	70%
	Female	56	30%
Mode of birth	C-section	54	31%
	NSVD	118	69%
Vaccination status	Up to date	91	51%
	Not completed	87	49%
Chronic illnesses	Present	89	50%
	Absent	90	50%
Treated in ER	Yes	167	94%
	No	10	6%
Admitted	Yes	125	71%
	No	52	29%
Route of administration	Oral	166	95%
	Intravenous	6	3%
	Intramuscular	3	2%
Administration of dexamethasone	Immediate	118	67%
	Late	59	33%
Early recovery	Recovered	53	31%
	Relapsed	119	69%

Relationship of independent variables with recovery from croup

In total, 53 (31%) patients recovered with a mean duration of illness of 2±1 days while those who relapsed were 119 (69%) patients with a mean duration of illness of 3±2 days. There was a significant difference in patients with chronic illnesses (85; 100%) between those who recovered (20; 24%) and those who relapsed (65; 76%), with a p-value of 0.04. For recovered patients (53; 100%), 50 (94%) patients were treated in the ER. Moreover, of these 53 patients, 40 (75%) patients were inpatient (IP) and those who were given dexamethasone immediately were 33 (62%) patients while those who were given late were 20 (38%). As for the relapsed patient (n=119), 111 (93%) patients were treated in ER. Moreover, of these 119 patients, 79 (66%) patients were IP and those who were given dexamethasone immediately were 79 (67%) patients while those who were given late were 39 (33%). Among those who were treated as IP, the presence of replacing symptoms was not associated with an increased demand for admissions (67% vs 75%) with x2=1.25, p-value= 0.26. Also, the mode of dexamethasone administration given early or late has no significant effect on the early recovery or relapse among our sample of patients with x2=0.35, p-value = 0.55 (Table 2).

**Table 2:** Relationship of independent variables with the recovery from croup (n=186) Dexa: dexamethasone, Kg: kilogram, cm: centimeters, NVSD: normal vaginal spontaneous delivery, ER: emergency room, N: number, SD: standard deviation, %: percentage *Chi-square/Fisher exact test, applied for categorical variables as applicable, significant at <0.05 ** t-test applied for numerical variables *** Relapsed: recurrent visits to ER with the same presentation and same clinical diagnosis

Variables	Categories	Early recovery				
		Recovered (N=53)		Relapsed (N=119) ***		p-value
		N(%)		N(%)		
Age in months**	Mean, SD	23	17	23	19	0.98
Duration of the illness (days)**	Mean, SD	2	1	3	2	0.38
Gender*	Male	37	31%	83	69%	0.99
	Female	16	31%	36	69%	
Mode of birth*	C-section	16	31%	36	69%	0.95
	NSVD	35	31%	77	69%	
Vaccination status*	Up to date	25	28%	63	72%	0.55
	Not completed	27	33%	56	68%	
Chronic illnesses*	Present	20	24%	65	76%	0.04
	Absent	33	38%	54	62%	
Treated in ER*	Yes	50	31%	111	69%	0.94
	No	3	30%	7	70%	
Admitted*	Yes	40	34%	79	66%	0.26
	No	13	25%	39	75%	
Route of administration	Oral	48	30%	111	70%	0.39
	Intravenous	2	33%	4	67%	
	Intramuscular	2	67%	1	33%	
Administration of dexamethasone	Immediate	33	29%	79	71%	0.55
	Late	20	34%	39	66%	

Dexamethasone use by immediate or late administration

There was no significant difference in recovery among patients who were given a dexamethasone dose immediately or late, with (67% vs 33%) of whom showed relapse with a p-value of 0.55. The preferred route of administration for immediate use was the oral route (p <0.001) while the intravenous or intramuscular route was given to only a few of the total patients, six (3%) and three (2%), respectively. Irrespective of the timing of administration of dexamethasone, 119 (69%) patients presented with relapse and required a subsequent dose of dexamethasone (Table 3).

**Table 3:** Dexamethasone use-related variables' distribution by immediate or late administration (N=186) Dexa: dexamethasone, ER: emergency room, N: number, SD: standard deviation, %: percentage *Fisher's exact test applied at a significance of <0.05 ϯ The chi-square statistic is significant at <0.05

Variables	Categories	Immediate N= 116		Late N= 51		All patients N= 186		
		N	%, SD	N	%, SD	N	%, SD	p-value
Treated in ER (n %)	Yes	116	69%	51	31%	167	94%	<0.001*
	No	2	20%	8	80%	10	6%	
Admitted (n %)	Yes	85	68%	40	32%	125	71%	0.56^ ϯ^
	No	33	63%	19	37%	52	29%	
Route of administration (n %)	Oral	111	68%	53	32%	166	95%	0.99
	Intravenous	4	67%	2	33%	6	3%	
	Intramuscular	2	67%	1	33%	3	2%	
Early recovery (n %)	Treated	33	62%	20	38%	53	31%	0.55^ ϯ^
	Relapsed	79	67%	39	33%	119	69%	

Predictors of recovery

The duration of illness was significantly associated with relapse among croup patients (OR=1.63; 95%CI: 1.13-2.35, p-value=0.01). Also, there was a significant association between patients who were treated in the ER and relapse (OR=11.36; 95%CI: 2.96-43.52, p-value=<0.001). See Table 4.

**Table 4:** Multiple logistic regression for predictors of relapse among patients with croup Variable(s) entered on step 1: Duration of the illness, Vaccination status, Chronic illnesses, Admitted, Administration of dexamethasone Significant results presented in bold vaccination reference: vaccinated Chronic illness those with illness present Admitted reference 'yes admitted' Dexamethasone dose given a reference 'immediate' Reference category as first (only for chronic illness last)

	Beta	S.E.	odds ratio	95% C.I.		P-value
				Lower	Upper	
Duration of illness (Days)	0.49	0.19	1.63	1.13	2.35	0.01
Vaccination status (Incomplete)	-0.15	0.47	0.86	0.34	2.16	0.75
Chronic illnesses (Present)	0.84	0.46	2.32	0.94	5.72	0.07
Admitted (Not)	2.43	0.69	11.36	2.96	43.52	<0.001
Administration of dexamethasone (Late)	-0.95	0.67	0.39	0.11	1.44	0.16

Dose-related recovery and relapse

There was no significant result related to dexamethasone dose between recovered and relapsed groups with a mean dose of 6.36 mg and 6.23 mg, respectively, and a mean repeated dose of 6.76 mg and 6.38 mg, respectively (Table 5).

**Table 5:** Dose-related recovered and relapsed

	Early recovery					
	Recovered			Relapsed		
	Mean	Standard Deviation	Mean		Standard Deviation	P-value
Dose of dexamethasone	6.36	2.22	6.23		2.64	.488
Dose if repeated	6.76	2.31	6.38		2.75	.460

## Discussion

Dexamethasone is effective in decreasing the length of stay (LOS), the use of epinephrine, visits, and/or (re)admissions that are required, as early as six hours in croup patients [13]. It has also been associated with more palatability, which was scored based on five degrees: 1 'dislike very much, 2 'dislike a little', 3 'not sure', 4 'like a little', and 5 'like very much’, and fewer side effects than other drugs that are being used to treat croup such as prednisolone [14-15]. In this study, we found that the difference between giving dexamethasone early (within 24 hours of signs and symptoms) or late is not significant neither for recovery nor relapse, but late administration is slightly better in recovery with 34% against the immediate with 30%, also slightly better in relapse with 60% against 71% for the immediate.

In another study that took place in Australia comparing dexamethasone (0.6 mg/kg), low-dose dexamethasone (0.15 mg/kg), and prednisolone (1 mg/kg), the type of oral steroid does not appear to have any clinically significant influence on efficacy, both acutely and for seven days following management [16-17].

Another study looked at the heliox treatment in patients with croup and found that in mild croup patients, heliox may not be more effective than 30% of humidified oxygen, but it may be beneficial in combination with dexamethasone for treating moderate croup patients. Also, heliox could be similar to 100% oxygen combined with one or two doses of adrenaline [18].

In case of admission, the difference between patients who were treated as inpatients and patients who were not is 9%; for those who were IP, they had more recovery with 34%; and for those who were not, the recovery was less with 25%. Also, those who were treated as IP had fewer relapses with 66% against 75% for those who were not. Moreover, an American study conducted in Colorado, found that among critically ill patients with croup, a significant number rebounded. Dexamethasone doses before pediatric intensive care unit (PICU) discharge did not predict whether they would rebound or the timing of their recovery [19].

Also, we found that there was a significant difference in patients with chronic illnesses in terms of recovery and relapse among 85 patients; those who recovered were 20 patients, which accounts for 24%, and the number of those who relapsed was 65 patients, which accounts for 77%. That gave us a p-value of (0.04), which indicates that chronic illnesses increase the incidents of relapse significantly, which is consistent with previous studies. However, there was no significant result related to the dexamethasone dose between recovered and relapsed groups with a mean dose of 6.36 mg and 6.23 mg, respectively, and a mean repeated dose of 6.76 mg and 6.38 mg, respectively.

In relation to the dose of dexamethasone despite whether the timing is immediate or late. We found an Australian study that showed that an oral dose of 0.15 mg/kg dexamethasone offered benefit to children with croup as early as 30 minutes after administration, with a lower croup score for children treated with dexamethasone [20].

In addition, we found a recent Canadian study that suggests there is no significant difference between the immediate or late administration of dexamethasone, but their result was not conclusive due to the low number of patients who received the medication of interest. Also, there were some differences between our study and their study in relation to some parts of the method, for example, the Canadian study included pediatric patients between six months and six years of age while our study included pediatric patients between three months and three years of age, which influenced the sample size for both studies. For our study, the sample size was 186, all between three months and three years of age. In contrast, the Canadian study sample size was 188, all between six months and six years of age. In another example, they excluded patients who took steroid therapy in the preceding two weeks or who had a prior visit to an ED due to croup in the past seven days while we did not. Also, in relation to the recording of early or late administration of dexamethasone, the Canadian study depended, for their data collection, on prehospital presentation and management, which were provided by the EMS through paper-based patient care records, ED presentation and management, and ED LOS, which were abstracted from paper-based hospital records while our study depended for data collection on the electronic medical record (Best Care) in the emergency department, King Abdullah Specialized Children's Hospital (KASCH), Riyadh, Saudi Arabia [14].

We acknowledge that our study has limitations. The study was conducted at a single center with a limited population. The research results may have been more generalizable if it was conducted in multiple centers with a larger population. In addition, due to the retrospective study design, no follow-up data of patients were obtained. Furthermore, the data were collected from electronic medical records, hence, there were missing data, for example, weight, height, and specific time of dose administration. Missing doses lead to the exclusion of many of the patients from the final analysis. Due to the definition of immediate or late administration, which was based on the duration of a whole day, we could not find a specific time when the dose was given and it was not documented in the system, which is one of the limitations that can affect the results. Therefore, the results should be interpreted cautiously in other settings.

## Conclusions

In conclusion, the difference between the early and late administration of dexamethasone in both recovery and relapse is not significant. Also, the presence of chronic illnesses is significantly affecting more relapses. Almost two-thirds of the children aged three months to three years needed admission after the initial assessment. Therefore, croup in children younger than five years is a common cause of admission. Moreover, there was no significant result related to the dexamethasone dose between the recovered and relapsed groups with a mean dose of 6.36 mg and 6.23 mg, respectively, and a mean repeated dose of 6.76 mg and 6.38 mg, respectively. Lastly, further prospective multicenter studies with a larger sample size are recommended.
